# Comparison of Transcriptome Profiles of the Fungus *Botrytis cinerea* and Insect Pest *Bradysia odoriphaga* in Response to Benzothiazole

**DOI:** 10.3389/fmicb.2020.01043

**Published:** 2020-06-12

**Authors:** Kaidi Cui, Yunhe Zhao, Leiming He, Jinfeng Ding, Beixing Li, Wei Mu, Feng Liu

**Affiliations:** ^1^Shandong Provincial Key Laboratory for Biology of Vegetable Diseases and Insect Pests, College of Plant Protection, Shandong Agricultural University, Tai’an, China; ^2^College of Plant Protection, Shandong Agricultural University, Tai’an, China

**Keywords:** benzothiazole, *Botrytis cinerea*, *Bradysia odoriphaga*, transcriptome, nutrient metabolism, melanogenesis, signaling pathway

## Abstract

Benzothiazole (BT) has a strong inhibitory effect on the growth and development of a wide spectrum of fungi and insects, such as *Botrytis cinerea* and *Bradysia odoriphaga*, that cause serious losses in agriculture. To investigate the underlying antifungal and insecticidal mechanisms of BT, RNA-seq analysis was performed for *B. cinerea* after BT treatment for 12, 24, and 48 h and for *B. odoriphaga* after BT treatment for 6 and 24 h. In *B. cinerea*, the pectin degradation process was inhibited, suggesting a low utilization of carbohydrate sources. As the treatment time was extended, the cell walls of *B. cinerea* thickened, and increases in melanin synthesis and ion transport were observed. In *B. odoriphaga*, signaling pathways including MAPK, insulin, adipocytokine, forkhead box class O, and peroxisome proliferator-activated receptor were activated at 6 h, and phosphoenolpyruvate carboxykinase was the core gene in the signal transduction pathways that responded to BT; digestive system and melanogenesis genes were obviously altered at 24 h. In addition, we identified several insecticidal target genes, such as trypsin, aminopeptidase N, and tyrosinase. Benzothiazole significantly affected nutrient metabolism, especially carbohydrate metabolism, in both species, and the pentose and glucuronate interconversions pathway was shared by both species, although the individual genes were different in each species. Overall, our results suggested that BT was a melanogenesis disrupter for the insect but an activator for the fungus. Our findings are helpful for deeply exploring the genes targeted by BT and for developing new pesticide compounds with unique mechanisms of action.

## Introduction

Benzothiazole (BT) is a volatile compound with a privileged bicyclic ring system that exists in many microorganisms (e.g., *Aspergillus clavatus* and *Bacillus subtilis*) (Seifert and King, 1982; Liu et al., 2009b) and natural plant products (e.g., grapes, tea leaves, and cranberries) (Ferrandino et al., 2012; Keri et al., 2015). It is known in the field of medicine that BT derivates have a wide spectrum of pharmacological activities (Ali and Siddiqui, 2013). Benzothiazole is a structure with an inherent affinity for different biological receptors and provides an ideal source of core scaffolds for targeted molecules in the design of new active compounds (Keri et al., 2015). Numerous therapeutic agents have been synthesized based on the BT nucleus, and this chemical family has attracted the attention of medicinal chemists. The primary results obtained in our research group have indicated that BT exhibits strong inhibition of a wide spectrum of fungi (e.g., *Botrytis cinerea, Fusarium oxysporum*, and *Colletotrichum capsici*) and insects (e.g., *Bradysia odoriphaga* and *Tribolium castaneum*) (Zhao et al., 2011). These results suggested that BT also has strong activity against fungi and pests in agriculture. Therefore, understanding the novel compound BT is important for synthesizing and developing other effective fungicides or insecticides.

Among the species that we tested with BT, the fungus *B. cinerea* and the insect *B. odoriphaga* were the most sensitive (Zhao et al., 2011). *B. cinerea* is the second most important plant pathogenic fungus in the world (Dean et al., 2012) and affects more than 200 plant species at the pre- and postharvest stages, resulting in massive losses during growth, transport, storage, and commercialization (Williamson et al., 2007). Benzothiazole has no cross-resistance with several fungicides currently used to control *B. cinerea*, such as carbendazim, procymidone, and pyrimethanil (Cui et al., 2017), suggesting that BT has a unique mechanism of action. *B. odoriphaga* is the most important pest attacking the Chinese chive (*Allium tuberosum*) and also affects other crops such as cabbage (*Brassica oleracea*), garlic (*Allium sativum*), and shallot (*Allium ascalonicum*) (Mei et al., 2003). Its larvae feed on roots, bulbs, and young stems, resulting in 30–80% production losses. Chen et al. (2014) verified that BT has strong fumigation activity against all developmental stages of *B. odoriphaga*. Considering that fruit and vegetable storage as well as soil fumigation may be possible applications of the volatile compound BT, choosing the pathogen *B. cinerea* and the insect *B. odoriphaga* as research subjects seemed suitable. Since BT has inhibitory activity against both *B. cinerea* and *B. odoriphaga*, the same target or mechanism of action of BT may be present in this pathogen and insect pest.

Although BT has useful antifungal and insecticidal activity, the unique mechanism of action of BT is largely unknown. Fortunately, next-generation sequencing (NGS)-based RNA-seq analysis provides efficient and rapid discovery of genes in the study of molecular biology (Schuster, 2008). Many genes involved in the inhibitory effects of exogenous compounds in fungi have been identified through transcriptome analyses (Chang et al., 2015; Lv et al., 2018; Zhao et al., 2018; Wang et al., 2019; Ren et al., 2020).

Uncovering the mechanism of action of BT is helpful for synthesizing similar active compounds in agricultural management of agricultural disease and pests. In the current study, we investigated the transcriptome responses of the fungus *B. cinerea* and the insect pest *B. odoriphaga* when exposed to BT, to identify their similarities and differences. Significantly changed pathways and potential target genes of BT were comprehensively analyzed, and the results will benefit the exploration of the potential mechanism underlying the antifungal and insecticidal activities of BT and the development of future novel pesticides.

## Materials and Methods

### Cultivation and Benzothiazole Treatment of *B. cinerea*

As described in our previous study, *B. cinerea* is highly sensitive to BT (Cui et al., 2017). An isolate, SX-9, was selected for the following study to explore the effect of BT on *B. cinerea*. The *B. cinerea* SX-9 isolate used in this study was collected from a pepper greenhouse in Shandong Province, China, and it was obtained by the single-spore method and maintained on potato dextrose agar (PDA; Difco^TM^ BD Diagnostics, Franklin Lakes, NJ, United States) plates. After a 24-h incubation period at 25°C in the dark (considered 0 h), *B. cinerea* isolate SX-9 was fumigated with 0.5 μL of BT (purity of 99%; Beijing Lark Technology Co., Ltd., Beijing, China) in 200 mL-volume fumigation plates, to create a final concentration of 2.5 μL/L (the concentration that caused 50% inhibition given 24 h of preincubation). For comparison, PDA plates without BT were used as controls. The mycelia were collected at three specific time points (12, 24, and 48 h) and quickly frozen in liquid nitrogen. Thus, six groups were subjected to RNA-seq, including the control (CON) at 12, 24, and 48 h and BT treatment at 12, 24, and 48 h. There were three biological replicates for each treatment, and mycelia collected from 10 PDA plates were pooled to form one biological replicate.

### Culture and Benzothiazole Treatment of *B. odoriphaga*

The culture of *B. odoriphaga* was previously described (Zhao et al., 2016a, b). The population of *B. odoriphaga* has been cultured in the laboratory since 2013. Fresh chive rhizomes in 1-cm pieces were used to rear the insects, which were maintained in Petri dishes at 25 ± 1°C with 70 ± 5% relative humidity and a 14:10 (light:dark) photoperiod. The newly emerged 4th instar larvae of *B. odoriphaga* were transferred from the Petri dishes and fumigated with the LC_30_ of BT (0.47 μL/L) in fumigation aquariums (10 L, 20 cm × 20 cm × 25 cm) at 25°C. After fumigation with BT for 6 h and 24 h, the live larvae in the BT and control groups were collected. Three biological replicates of 20 larvae each were frozen in liquid nitrogen.

### Transmission Electron Microscopy (TEM) of *B. cinerea*

Transmission electron microscopy (TEM) was carried out to investigate the mechanism of action of BT on the ultrastructure of *B. cinerea*. Hyphae samples were collected from the edges of control and BT-treated *B. cinerea* colonies. The hyphae were fixed with 2.5% glutaraldehyde in 0.1 M sodium cacodylate buffer (pH 7.4) for 2 h at 4°C. After rinsing in the same buffer several times, the samples were postfixed in 1% OsO_4_ in the same buffer for 2 h at 4°C, dehydrated through an ethanol series, and embedded in Epon-Araldite resin. Thick sections were prepared with an LKB ultramicrotome, stained with uranyl acetate and lead citrate, and viewed under a JEOL 1200EX transmission electron microscope (JEOL, Tokyo, Japan). The thickness of the cell wall was also measured and recorded. Due to the complexity of the larval body and the lack of a predicted specific site of action of BT, we did not observe *B. odoriphaga* by TEM.

### RNA Extraction, Library Preparation and Sequencing

Total RNA was extracted from frozen *B. cinerea* mycelial samples using an E.Z.N.A. Fungal RNA Kit (Omega, Norcross, GA, United States). Total RNA was extracted from *B. odoriphaga* samples using TRIzol reagent (Invitrogen, Grand Island, NY, United States) and treated with DNase I. The RNA integrity value was confirmed with an Agilent 2100 Bioanalyzer (Agilent, Santa Clara, CA, United States). The concentration and purity of the RNA were determined with a NanoDrop 2000 (Thermo Fisher Scientific, Waltham, MA, United States). According to the manufacturer’s (Illumina) protocol, poly-(A) messenger RNA (mRNA) was isolated using oligo-(dT) magnetic beads and fragmented into short pieces using fragmentation buffer. Random hexamer primers were used to synthesize cDNA. After purification for terminal repair and adaptor ligation, the products were amplified through PCR to create cDNA libraries. Then, the *B. cinerea* and *B. odoriphaga* libraries were sequenced on a BGISEQ-500 platform and an Illumina HiSeq^TM^ 4000 platform, respectively, at the Beijing Genomics Institution (BGI, ShenZhen, China).

### RNA-Seq Data Processing and Identification of Differentially Expressed Genes

For the *B. cinerea* transcriptome, the raw reads from the sequencing machine were cleaned by filtering out the adaptor reads, low-quality reads (bases with quality value ≤5 accounting for more than 50% of the read), and reads with unknown bases accounting for more than 10% of the read. The clean reads were saved in FASTQ format and then mapped to the *B. cinerea* B05.10 genome database using HISAT (Kim et al., 2015) with no more than two base mismatches. The gene expression level was normalized and estimated by FPKM (fragments per kilobase of transcript per million mapped reads) (Sims et al., 2014). Differentially expressed genes (DEGs) were screened between the CON and BT treatments at each time point using the NOISeq method (Tarazona et al., 2011). Genes with a combination of probability ≥ 0.8, FDR ≤ 0.001 and absolute value of log_2_ ratio ≥ 1 were considered DEGs.

For the *B. odoriphaga* transcriptome, the raw reads from the sequencing machine were cleaned by filtering out the adaptor reads, low-quality reads (bases with quality value ≤15 accounting for more than 20% of the read), and reads with unknown bases accounting for greater than 5% of the read. Trinity software (Grabherr et al., 2011) was used to perform *de novo* assembly with the clean reads, and the TGICL package (Pertea et al., 2003) was used to cluster the transcripts into unigenes. To obtain a comprehensive functional annotation, unigenes were determined using the BLAST program (Altschul et al., 1990) by querying against public functional databases as follows: National Center for Biotechnology Information (NCBI) non-redundant nucleotide sequences (NT), NCBI non-redundant protein sequences (NR), Clusters of Orthologous Groups of proteins (COG), Kyoto Encyclopedia of Genes and Genomes (KEGG) and Swiss-Prot. Blast2GO (Conesa et al., 2005) with NR annotation was used to obtain the Gene Ontology (GO) annotation and InterProScan 5 (Quevillon et al., 2005) was used to obtain the InterPro annotations. Bowtie2 (Langmead and Salzberg, 2012) was used to map the clean reads to the assembled unigenes, and RSEM (Li and Dewey, 2011) was used to obtain the read count for each unigene in each sample. The gene expression level was normalized and estimated by FPKM. Differentially expressed genes were screened between the CON and BT treatments at each time point using DESeq2 (Love et al., 2014). Genes with both an absolute value of log_2_ ratio ≥ 1 and an adjusted *P*-value ≤ 0.05 were considered DEGs.

### GO and KEGG Enrichment Analyses of the DEGs

The Blast2GO program was used to assign all the DEGs to GO categories, and WEGO software (Ye et al., 2006) was used to perform functional classification of GO terms. In addition, GO (Ashburner et al., 2000) and KEGG (Kanehisa and Goto, 2000) pathway enrichment analyses were performed on the DEGs. Gene Ontology terms with a threshold-corrected *P*-value ≤ 0.05 were considered significantly enriched in DEGs, and KEGG pathways that fulfilled the criterion of *Q*-value ≤ 0.05 were defined as significantly enriched.

### Effects of Benzothiazole on Melanin Biosynthesis in *B. cinerea*

In the fumigation assay, we found, by accident, that BT may accelerate the melanization of *B. cinerea*. Therefore, we performed a series of tests to verify this phenomenon and investigated whether this effect could affect mycelial growth and virulence. Mycelial plugs (5 mm) from the edges of 3-day-old colonies were inoculated on the centers of PDA plates with or without tricyclazole, an inhibitor of fungal dihydroxynaphthalene (DHN) melanin biosynthesis (Zhang et al., 2015). We set three concentrations of tricyclazole at 0, 10 and 50 μg/mL. Beyond that, 0.5 or 1 μL of BT (final concentration of 2.5 or 5 μL/L) was added to each plate to build a fumigation environment. Plates without BT and tricyclazole were used as controls. Every treatment consisted of six parallel samples. The diameter of each mycelium colony was measured after two days, and a photograph of *B. cinerea* colony melanization was observed and recorded. Then, mycelium plugs from the edge of each *B. cinerea* colony in each treatment were inoculated on detached cucumber leaves (one plug per leaf) to verify the role of melanin in the virulence of *B. cinerea*. The infected leaves were maintained in a container with 85% relative humidity at 25°C with a photoperiod of 12 h. The lesion diameter minus 5 mm (the diameter of the mycelium plug) was considered the lesion growth diameter. There were six cucumber leaves for each treatment. In addition, we identified the expression levels of seven melanin biosynthesis-related genes using reverse transcription quantitative PCR (RT-qPCR).

### RT-qPCR Validation

Gene-specific primers for RT-qPCR were designed using the Beacon Designer 7.0 program, and their sequence information is listed in Supplementary Table S1. Total RNA was extracted from frozen *B. cinerea* mycelia and *B. odoriphaga* larvae samples using an E.Z.N.A. Fungal RNA Kit and TRIzol reagent (Invitrogen, Grand Island, NY, United States), respectively. Then, 1 μg of RNA for each sample was used to synthesize first-strand cDNA using the TransScript All-in-One First-Strand cDNA Synthesis SuperMix for qPCR Kit (Transgen Biotech, Beijing, China). Reverse transcription quantitative PCR analysis was performed using TransStart^®^ Top Green qPCR SuperMix (TransGen Biotech, Beijing, China) on a Light Cycler 96 system (Roche, Basel, Switzerland). Each 20 μL reaction mixture contained 10 μL of 2 × TransStart^®^ Top Green qPCR SuperMix, 0.4 μL of each forward and reverse primer, 8.2 μL of double distilled water and 1 μL of cDNA. The cycling program was 94°C for 30 s, followed by 45 cycles at 94°C for 5 s, 56°C for 15 s and 72°C for 10 s. There were three biological replicates and three technical replicates for each gene of each sample. β-tubulin and ribosomal protein S15 (RPS15; Shi et al., 2016) were used as the internal control genes for the RT-qPCR analyses of *B. cinerea* and *B. odoriphaga*, respectively. The relative gene expression level in each comparison between a BT-treated sample and its control was calculated using the 2^– ΔΔCt^ method (Livak and Schmittgen, 2001). The statistical analysis was performed with one-way ANOVA followed by Tukey’s HSD method (*P* < 0.05) via SPSS 18.0 software (SPSS Inc., Chicago, IL, United States).

## Results

### Effects of Benzothiazole on the Ultrastructure of *B. cinerea*

**FIGURE 1:**
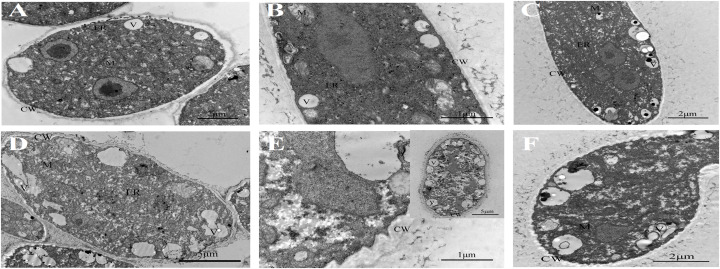
Transmission electron micrographs of *Botrytis cinerea* with or without benzothiazole treatment. **(A–C)** Untreated *B. cinerea* at 12, 24, and 48 h. Many organelles were observed, including the cell wall (CW), vacuole (V), mitochondria (M) and endoplasmic reticulum (ER). **(D–F)** *B. cinerea* hyphae after fumigation with benzothiazole for 12, 24, and 48 h, showing many anomalous vacuoles, significant thickening of the cell wall, and indistinguishable organelles in the cytoplasm.

**FIGURE 2:**
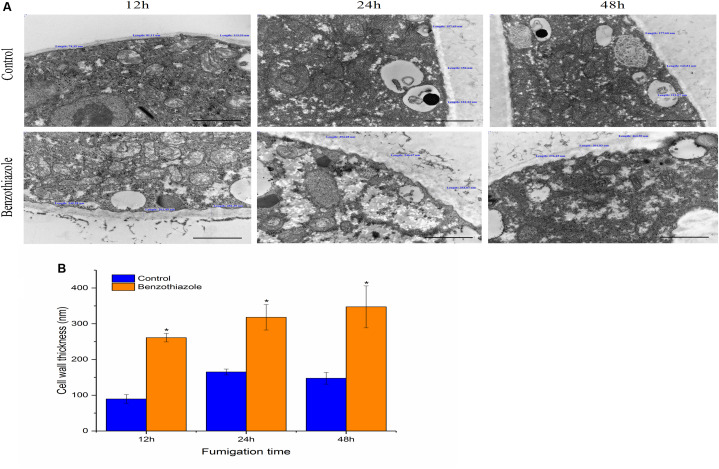
Effects of benzothiazole on the cell wall thickness of *Botrytis cinerea* (scale bar = 1 μm). **(A)** High magnification of the cell wall of *B. cinerea* showing thickness measured at three different positions of the cell wall. **(B)** Statistical analysis of the cell wall thickness of benzothiazole-treated and control *B. cinerea*. The asterisk indicates a significant difference from the respective control.

Untreated *B. cinerea* showed normal organelles, including regularly shaped mitochondria and rough endoplasmic reticulum (Figures 1A–C). When *B. cinerea* was treated with 2.5 μL/L BT for 12 h, some large, anomalous vacuoles were observed (Figure 1D). As the fumigation time was prolonged, the vacuolization increased, and the organelles became indistinguishable in the cytoplasm (Figures 1E,F). Most obviously, greatly increased cell wall thickness was observed in the BT-treated samples, and there was a positive correlation between fumigation time and cell wall thickness (Figure 2).

### RNA-Seq Analysis of the *B. cinerea* Transcriptome

Six libraries with three biological replicates per treatment were constructed. The RNA-seq analysis data were deposited in the NCBI Sequence Read Archive (SRA) (SRP174462). Approximately 23 million clean reads were generated per sample, with >86% of reads mapping to the *B. cinerea* B05.10 genome, and an average of 81% of these reads were matched uniquely (Supplementary Table S2). The expression levels of 15,451 genes were calculated using the FPKM method.

#### Identification of DEGs in *B. cinerea*

**FIGURE 3:**
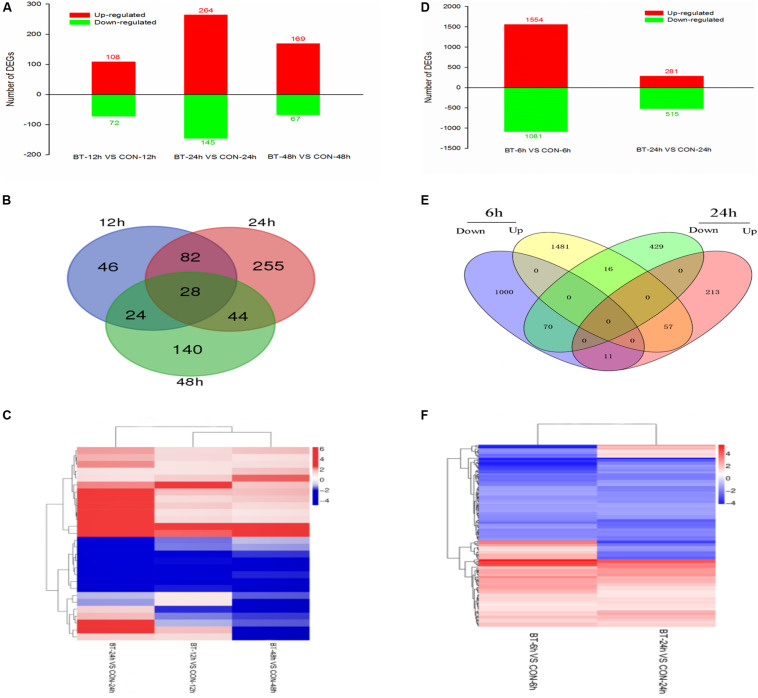
Gene expression profiling of the **(A–C)** *Botrytis cinerea* and **(D–F)** *Bradysia odoriphaga* transcriptomes. **(A,D)** Numbers of DEGs in each comparison group. **(B,E)** Venn diagram showing the shared and specific DEGs (fold change >2) at each time point. **(C,F)** Heatmap of common DEGs.

DEGs were screened with the criteria |log2 ratio| ≥ 1 and probability ≥ 0.8 when treated samples were compared to their respective controls (Supplementary Table S3). First, the maximum number of DEGs was observed at 24 h, suggesting that at the mRNA level, 24 h was the most acute time point in the response process (Figure 3A). Comparing the treatment to the control group at 12 h (CON-12 h vs BT-12 h), 180 DEGs were identified in the BT-treated group, with 108 upregulated and 72 downregulated genes; comparing BT-24 h with CON-24 h, 409 DEGs (264 upregulated and 145 downregulated) were generated. At the longest exposure time (CON-48 h vs BT-48 h), the number of DEGs was decreased; of these DEGs, 169 and 67 genes were upregulated and downregulated, respectively (Figure 3A and Supplementary Table S3). The Venn diagram in Figure 3B shows the commonly and differentially expressed DEGs at the three treatment time points. Twenty-eight core sets of DEGs (Figure 3B) were used for clustering analysis (Figure 3C), among which 13 and eight genes were consistently upregulated and downregulated during the time series transcriptome profiling, respectively (Supplementary Table S4). These 21 genes were considered important potential target genes associated with BT response in *B. cinerea*. However, as many of these 28 core DEGs encoded predicted or hypothetical proteins, no process or pathway analysis was performed.

#### GO and KEGG Enrichment Analyses of the DEGs in *B. cinerea*

**FIGURE 4:**
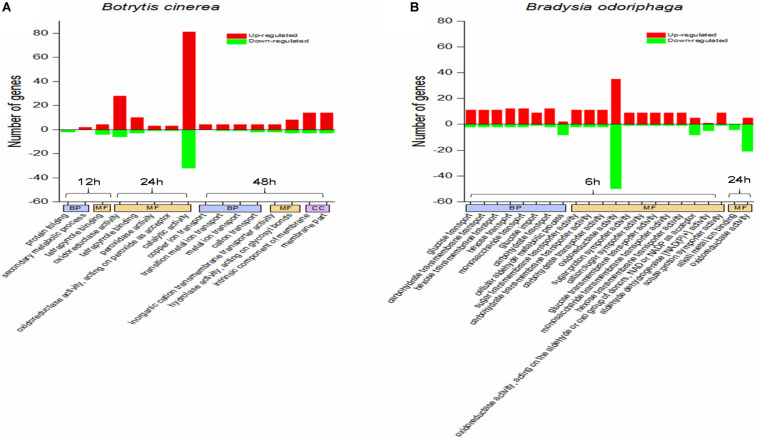
GO term enrichment analysis for the DEGs in panels **(A)** *Botrytis cinerea* and **(B)** *Bradysia odoriphaga.* GO categories are functionally classified into biological process (BP), molecular function (MF), and cellular component (CC). The GO terms in the same category at the same time point are arranged from left to right in ascending order of *P*-values.

Figure 4A shows the significantly enriched GO terms (corrected *P*-value < 0.05). In the comparison CON-12 h vs BT-12 h, the DEGs in the biological process (BP) category were enriched in the “protein folding” (downregulated genes) and “secondary metabolic process” (upregulated genes) terms, and the main molecular function (MF) term was “tetrapyrrole binding.” At 24 h, the highly enriched MF terms were “oxidoreductase activity” and “catalytic activity.” At 48 h, obvious morphological changes had occurred, and the DEGs were mostly enriched in BP terms related to ion transport, such as “copper ion transport,” “transition metal ion transport,” “metal ion transport” and “cation transport,” The significant MF terms were “inorganic cation transmembrane transporter activity” and “hydrolase activity, acting on glycosyl bonds,”

**FIGURE 5:**
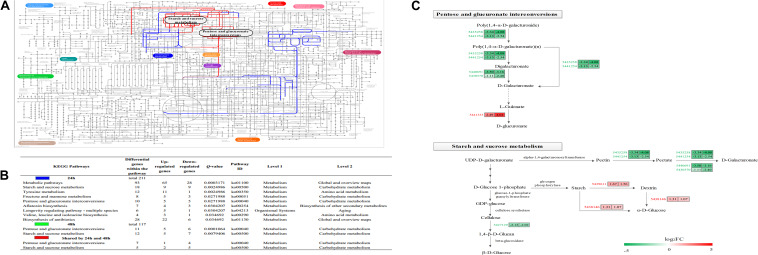
Characterization of the *Botrytis cinerea* transcriptome. **(A)** Projection of the *Botrytis cinerea* transcriptome on the KEGG pathways using iPath (Letunic et al., 2008). Significant pathways at 24 and 48 h are shown in blue and green, respectively. Red lines correspond to shared pathways at 24 and 48 h. Note: Green lines are covered by red lines. **(B)** Detailed information about the KEGG enrichment analysis of the DEGs. **(C)** Abundance patterns of the genes participating in the shared pathways. Annotations of the genes are available in Supplementary Table S5.

The KEGG pathway enrichment analysis results showed that 0, 9 and 2 pathways were significantly enriched at 12, 24, and 48 h, respectively (Figure 5B), while two pathways (pentose and glucuronate interconversions, starch and sucrose metabolism) were enriched at both 24 and 48 h (Figures 5A,B). There were eight common genes in the shared pathways, and the expression abundance of these genes is shown in Figure 5C and Supplementary Table S5. Interestingly, two polygalacturonases (*Bcpgx1*, *Bcpg1*) and two pectin methylesterases (PMEs; *Bcpme1*, *Bcpme2*) belonged to both the “pentose and glucuronate interconversions” and “starch and sucrose metabolism” pathways; these genes participated in pectin degradation and were downregulated in the treatment group.

### Increased Melanin Biosynthesis and Decreased Virulence in *B. cinerea* Under Benzothiazole Treatment

**FIGURE 6:**
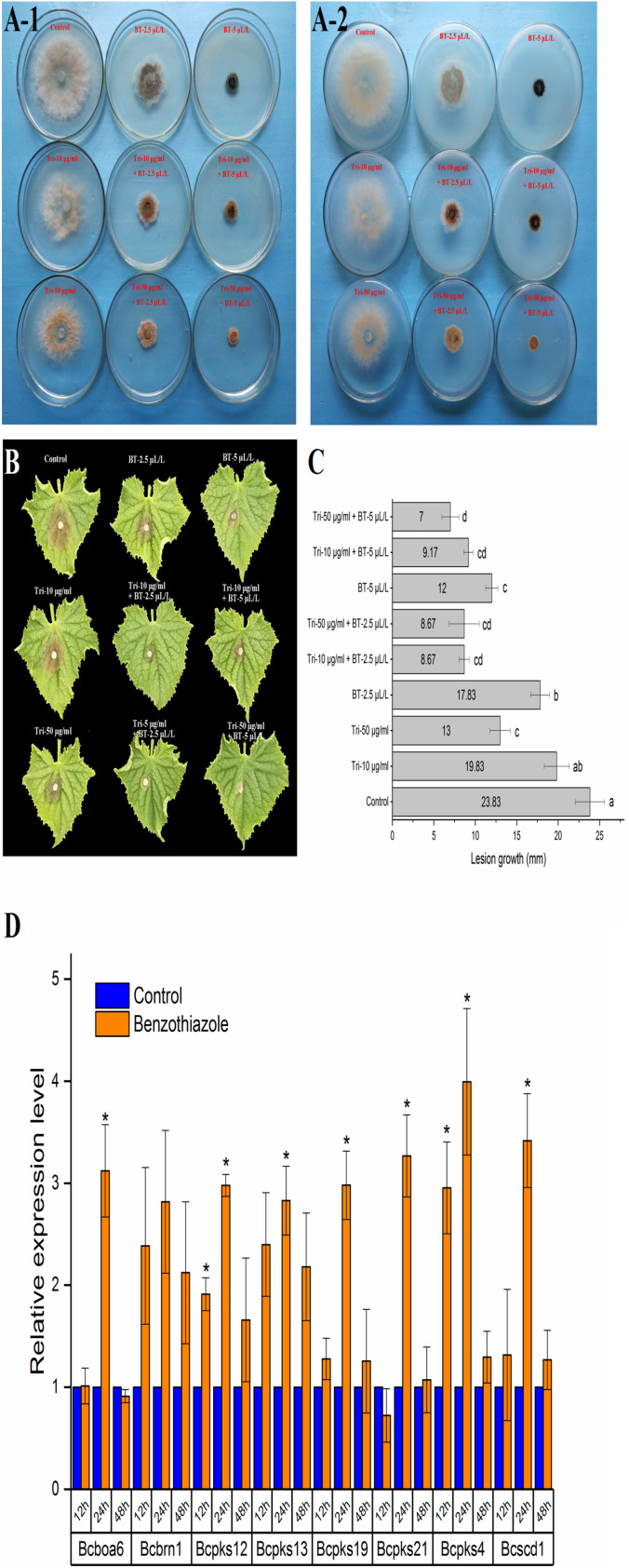
Analysis of the effect of benzothiazole on melanin production in *Botrytis cinerea*. **(A)** Melanin biosynthesis of *Botrytis cinerea* with or without exposure to benzothiazole and tricyclazole (a fungal dihydroxynaphthalene melanin biosynthesis inhibitor) observed from the front **(A-1)** and back **(A-2)** of the petri dishes. **(B)** Virulence variation resulting from different extents of melanin biosynthesis inhibition. **(C)** Lesion growth diameters on the cucumber leaves from panel **(B)**. **(D)** Relative expression levels of melanin biosynthesis-related genes in *Botrytis cinerea* after fumigation with benzothiazole. The asterisk indicates a significant difference from the respective control.

As shown in Figure 6A, BT could stimulate *B. cinerea* to produce black pigment, and the DHN melanin synthesis inhibitor tricyclazole induced orange pigment production in the fungi, indicating that the black pigment was melanin. Melanin in fungi is considered protective and plays a role in fungal pathogenesis; however, we found that even with the increase in melanin, *B. cinerea* was less virulent after BT treatment (Figure 6B). Moreover, the addition of tricyclazole decreased the lesion growth of *B. cinerea* (Figures 6B,C). Based on the RT-qPCR results, we verified that the expression of melanin synthesis-related genes was increased (Figure 6D).

### RNA-Seq Analysis of the *B. odoriphaga* Transcriptome

#### Illumina Sequencing, *de novo* Assembly, and Functional Unigenes Annotation

As *B. odoriphaga* does not have a reference genome, the *B. odoriphaga* transcriptome was sequenced with the Illumina HiSeq platform, and *de novo* assembly was performed. Four libraries from 4th instar larvae of *B. odoriphaga* with or without BT treatment were sequenced. The Q20 (error probability of 0.01) of the clean reads for each sample was higher than 98% (Supplementary Table S6). After the *de novo* assembly, a total of 63,046 unigenes were generated, with a total length of 76,377,590 bp, a mean length of 1,211 bp, an N50 length of 2,091 bp, and a GC content of 37.69% (Supplementary Table S6). The raw sequencing data were deposited in the NCBI SRA database under the accession number SRP174932.

A total of 63,046 unigenes were annotated against seven public databases with an E-value cutoff of 10^–5^. As shown in Supplementary Table S7, 35,945 unigenes (57.01%) were annotated in at least one of the Nr, Nt, Swiss-Prot, COG, InterPro, KEGG, and GO databases. The number of unigenes annotated in specific or shared databases is presented in Supplementary Figure S1, which shows that 12,631 unigenes were annotated in all five main databases (NR, InterPro, Swiss-Prot, COG, and KEGG). The Nr database (52.68%) showed the highest sequence similarity (Supplementary Table S7). Based on the Nr annotations, the homologous species distribution is shown in Supplementary Figure S2. The sequences of *B. odoriphaga* were highly similar to the sequences of *Aedes aegypti* (10.34%), *Aedes albopictus* (7.39%), *Culex quinquefasciatus* (6.50%), *Anopheles gambiae* str. PEST (4.14%), *Anopheles sinensis* (3.40%) and *Anopheles darlingi* (2.63%).

#### Detection of DEGs in *B. odoriphaga*

Constitutive differential expression (| log2(fold change) | ≥ 1, adjusted *P*-value ≤ 0.05) was apparent between the BT and CON groups; 1,554 upregulated and 1,081 downregulated unigenes were identified at 6 h, and 281 upregulated and 515 downregulated unigenes were identified at 24 h (Figure 3D and Supplementary Table S8). Similar to the *B. cinerea* transcriptome results, a higher number of upregulated genes was detected. A total of 154 unigenes were shared by the 6 and 24 h-treatment groups, with 57 and 70 unigenes commonly upregulated and downregulated, respectively, at both time points (Figure 3E). The intersection heatmap of 154 DEGs is shown in Figure 3F.

#### Functional Enrichment Analysis of DEGs in *B. odoriphaga*

It was obvious that BT fumigation for 6 h mainly activated BP terms associated with carbohydrate transport, including glucose transport, carbohydrate transmembrane transport, hexose transmembrane transport, hexose transport, monosaccharide transport, glucose import, and carbohydrate transport. As the duration increased to 24 h, the DEGs involved in the MF terms alkali metal ion binding and oxidoreductase activity were mainly downregulated (Figure 4B).

**FIGURE 7:**
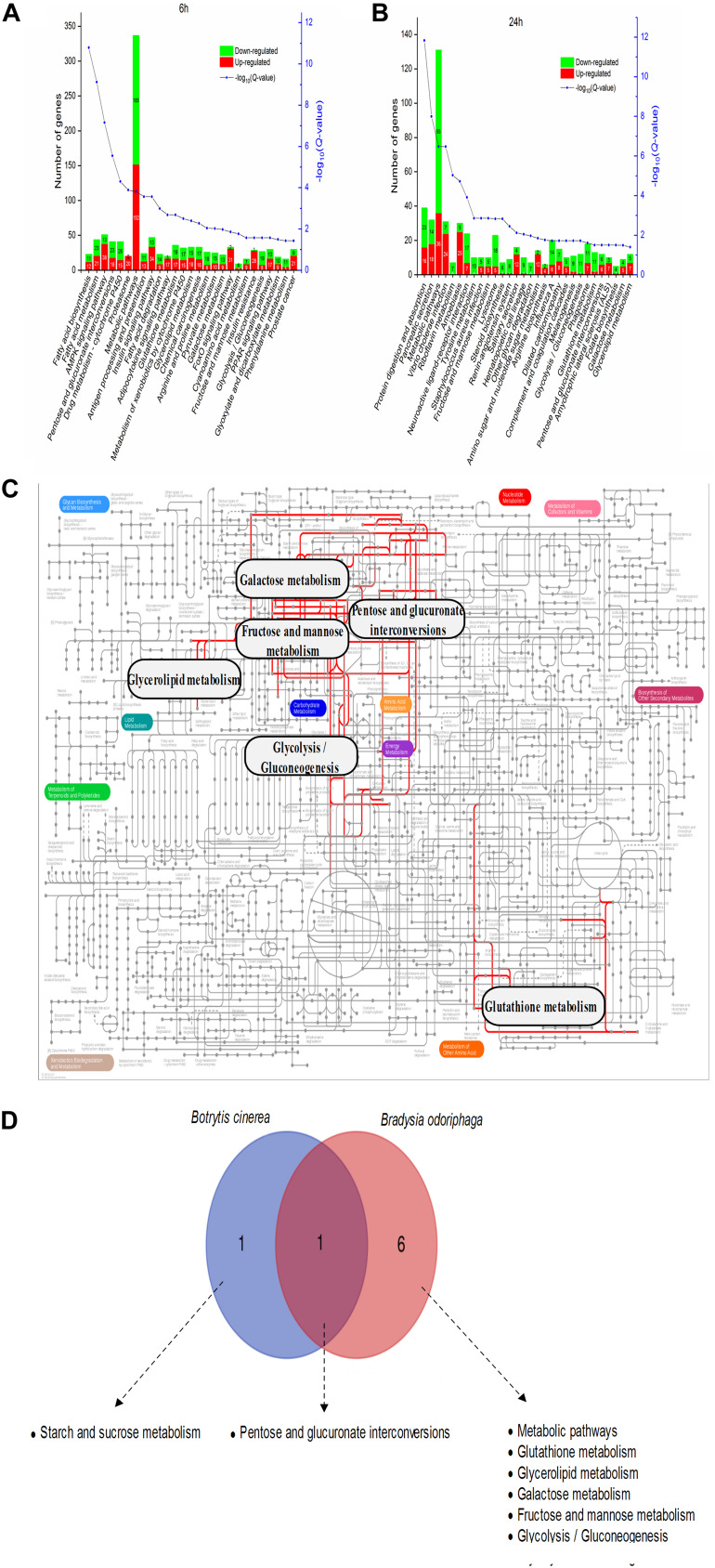
Metabolic pathway analysis of the DEGs in *Bradysia odoriphaga*. **(A,B)** KEGG enrichment analysis of the DEGs in *Bradysia odoriphaga* in response to benzothiazole for 6 and 24 h. **(C)** Projection of the *Bradysia odoriphaga* transcriptome on the KEGG pathways using iPath (Letunic et al., 2008). The common significantly changed pathways shared in the 6- and 24-h groups are shown with red lines. **(D)** Shared and different pathways between *Botrytis cinerea* and *Bradysia odoriphaga*. A Venn diagram was drawn using the data from Figure 5B (common pathways shared at 24 and 48 h in *Botrytis cinerea*) and Figure 7B (common pathways shared at 6 and 24 h in *Bradysia odoriphaga*).

As shown in Figure 7A and Supplementary Table S9, 6 h of BT treatment activated many pathways involved in signal transduction and the endocrine system, including the AMP-activated protein kinase (AMPK) signaling pathway (51 DEGs; 38 upregulated, 13 downregulated), insulin signaling pathway (47; 34, 13), adipocytokine signaling pathway (20; 17, 3), Forkhead box class O (FoxO) signaling pathway (33; 31, 2), and (peroxisome proliferator-activated receptor) PPAR signaling pathway (30; 17, 13). Notably, phosphoenolpyruvate carboxykinase (PEPCK) is a core gene participating in all five of these signaling pathways (Supplementary Figure S3). The six most enriched pathways were “metabolic pathways” (337 DEGs), “AMPK signaling pathway” (51), “insulin signaling pathway” (47), “fatty acid metabolism” (44), “pentose and glucuronate interconversions” (41), and “drug metabolism – cytochrome P450” (41). After a longer fumigation of 24 h, most of the DEGs were enriched in “metabolic pathways,” “protein digestion and absorption” and “pancreatic secretion” (Figure 7B). It was obvious that “glycolysis/gluconeogenesis,” “glutathione metabolism,” “tyrosine metabolism” and “melanogenesis” were mostly associated with downregulated proteins. In addition, the salivary secretion of *B. odoriphaga* was increased. From the genes participating in each KEGG pathway (Supplementary Table S9), we can see that the associated digestive enzymes, including trypsin, chymotrypsin, carboxypeptidase and α-amylase, were significantly altered. It is worth noting that there were 5 upregulated and 11 downregulated genes encoding trypsin, and these genes comprised a large proportion of the DEGs belonging to the pathways “protein digestion and absorption” (16 out of 39), “pancreatic secretion” (16 out of 32), “neuroactive ligand-receptor interaction” (16 out of 24) and “influenza A” (16 out of 30). In addition, 8 aminopeptidase N (APN) genes participating in the “glutathione metabolism” pathway (8 out of 13) were downregulated. Seven downregulated genes encoding tyrosinase represented a large proportion of the DEGs belonging to the pathways “tyrosine metabolism” (7 out of 10) and “melanogenesis” (7 out of 11).

Seven pathways were enriched at both 6 and 24 h: “pentose and glucuronate interconversions,” “metabolic pathways,” “glutathione metabolism,” “glycerolipid metabolism,” “galactose metabolism,” “fructose and mannose metabolism,” and “glycolysis/gluconeogenesis” (Figure 7C).

### Pathways Induced in Both *B. cinerea* and *B. odoriphaga* in Response to Benzothiazole

“Pentose and glucuronate interconversions” were significantly enriched in both the *B. cinerea* and *B. odoriphaga* transcriptomes (Figure 7D).

### RT-qPCR Validation of Benzothiazole-Responsive Genes

**FIGURE 8:**
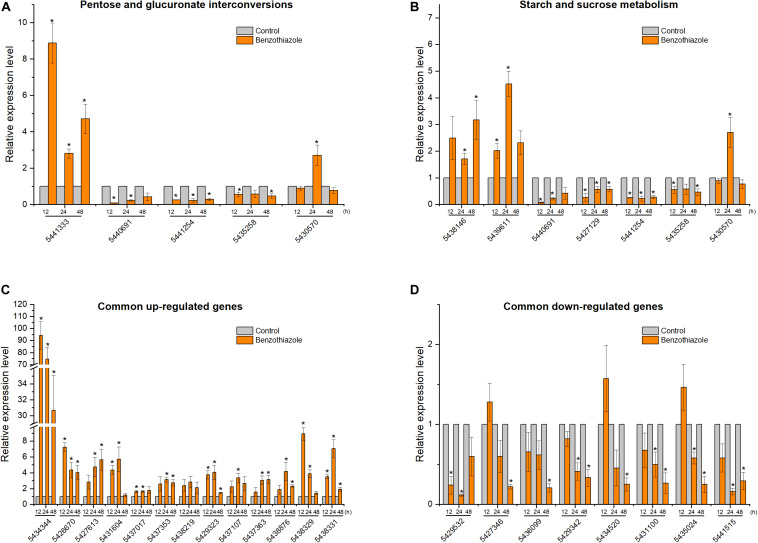
Relative expression levels of selected DEGs in *Botrytis cinerea* by RT-qPCR. **(A)** Expression of genes participating in the pentose and glucuronate interconversions pathway. **(B)** Expression of genes participating in the starch and sucrose metabolism pathway. **(C,D)** Expression of 13 common upregulated and 8 common downregulated genes at the three time points (12, 24, and 48 h). β-tubulin was used as an internal control gene for normalization. The asterisk indicates a significant difference from the respective control.

**FIGURE 9:**
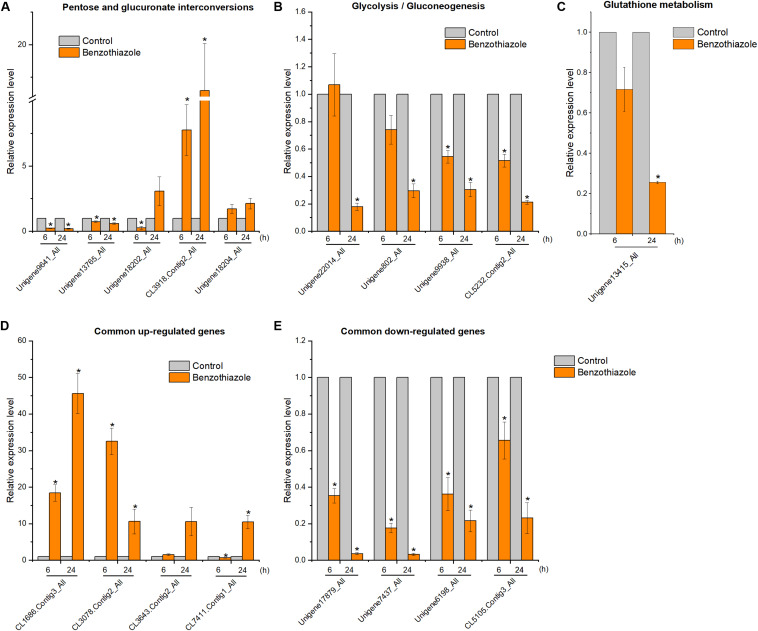
Relative expression levels of selected DEGs in *Bradysia odoriphaga* by RT-qPCR. **(A)** Expression profiles of genes related to the pentose and glucuronate interconversions pathway. **(B)** Expression profiles of genes related to the glycolysis/gluconeogenesis pathway. **(C)** Expression profiles of genes related to the glutathione metabolism pathway. **(D,E)** Expression profiles of 4 common upregulated and 4 downregulated DEGs at 6 and 24 h. Ribosomal protein S15 (RPS15) was used as an internal control gene for normalization. The asterisk indicates a significant difference from the respective control.

After BT treatment, 36 and 18 genes (Supplementary Table S1) for *B. cinerea* and *B. odoriphaga*, respectively, were chosen for RT-qPCR validation. Although the exact fold change ratio for the expression of each gene between the RT-qPCR and RNA-seq data was different, they showed consistent trends (Figures 8, 9). This result suggested that the RNA-seq and RT-qPCR results presented high consistency.

## Discussion

### Thickened Cell Walls and Increased Melanin Biosynthesis in *B. cinerea* After Benzothiazole Treatment

At the ultrastructural level, increased cell wall thickness was the most significant change caused by BT, and this change presented a concentration-responsive profile. The cell wall is the first protective barrier of *B. cinerea*, and this increase in cell wall thickness may be a defense response. DHN melanin is a crucial component of the extracellular matrix of *B. cinerea* (Zhang et al., 2015), and melanin granules are localized to the cell wall (Eisenman and Casadevall, 2012). Benzothiazole increased melanin biosynthesis, and it can reasonably be speculated that this increase in melanin synthesis may play a role in the thickening of the cell wall. However, this hypothesis requires further in-depth verification of whether there is an inevitable connection between these two processes. We have shown that inhibition of melanin attenuates *B. cinerea* infection, proving the importance of melanin in *B. cinerea* infection. Interestingly, the accumulation of melanin did not necessarily increase virulence. It has been reported that genes related to melanin synthesis, such as *Bcpks13*, *Bcbrn1* and *Bcscd1*, participate in the melanization of *B. cinerea* but negatively regulate its virulence (Zhang et al., 2015). Therefore, we investigated seven melanin-related genes (including *Bcpks13*, *Bcbrn1* and *Bcscd1*) to verify their expression levels, and the RT-qPCR results showed that these genes showed a trend toward increased expression. In addition, copper plays an important role in melanin production, likely because copper works as a required cofactor for melanin biosynthetic enzymes (Eisenman and Casadevall, 2012). Consistent with this role of copper, in the RNA-seq analysis of *B. cinerea*, we found that the copper ion transporter process was significantly different in the 48 h fumigation group. All the results showed that BT led to melanin accumulation at both the phenotype and transcript levels but negatively regulated the virulence of *B. cinerea*. Melanin is multifunctional and can improve fungal survival ability under a wide range of environmental stresses (Toledo et al., 2017). Considering that melanin accumulation in the fungal cell wall can prevent solute efflux (Liu et al., 2016), it is reasonable to speculate that melanin could avoid or reduce nutrient leakage in *B. cinerea*. In that case, however, why did the increase in melanin inhibit the growth and virulence of *B. cinerea*? We speculate that the production of melanin, a secondary metabolite, consumes many metabolic resources, thereby interfering with other normal cellular growth and development processes.

### Potential Target Genes Associated With Benzothiazole in *B. cinerea*

The eight commonly downregulated genes were all hypothetical or predicted genes, which made understanding their functions challenging. Nevertheless, among them, two genes (GeneID: 5,427,346 and 5,435,024) had descriptions in the KEGG database and conserved domain descriptions on NCBI, which helped us to predict their functions in response to the chemical compound BT. The hypothetical protein BC1G_14644 (5,427,346) has a conserved domain of the SIR2 superfamily of proteins, which includes silent information regulator 2 (Sir2) enzymes^1^. Sir2 proteins (sirtuins) act as NAD^+^-dependent deacetylases in all eukaryotes; with NAD^+^ as a cofactor, they catalyze the conversion of acetylated lysine to deacetylated lysine, nicotinamide and 2’-O-acetyl-ADP-ribose (OAADPr) (Smith and Denu, 2006). Silent information regulator 2 NAD^+^-dependent protein deacetylases display a variety of physiological functions, such as transcriptional silencing (Gasser and Cockell, 2001; Rusche et al., 2003), apoptosis (Luo et al., 2001), mitosis (Dryden et al., 2003), fatty acid metabolism (Starai et al., 2002), and lifespan extension (Tissenbaum and Guarente, 2001; Rogina and Helfand, 2004). Thus, the observed decrease in BC1G_14644 transcription suggested a disruption of the above cellular processes and a shortened lifespan in *B. cinerea*. Another hypothetical protein, BC1G_07058 (5,435,024), contains a conserved domain of glycosyl hydrolase family 71, which is a family of α-1,3-glucanases. Fungal α-1,3-glucanases attack the plant cell wall by hydrolyzing α-1,3-glucan, and the observed decrease in α-1,3-glucanase suggested weakened virulence after BT treatment, consistent with the result seen in Figure 6B.

Among the commonly increased genes, the gene 5,434,344 (ATP-binding cassette, subfamily G, member 2, SNQ2) showed the highest expression during exposure to BT, and it was the only ABC transporter gene detected in our study. It is well known that ATP-binding cassette subfamily G member 2 (ABCG2) proteins can act on a broad array of xenobiotic compounds as substrates (Kusuhara and Sugiyama, 2007), and the ABC transporter plays an important role in the energy-dependent efflux of fungicides in fungi (Andrade et al., 2000; Hayashi et al., 2001). The rapid and high increase in the expression of this ABC transporter could decrease the accumulation of BT in *B. cinerea*. We also identified an increase in cytochrome P450 (gene 5,437,017), which plays an important role in the detoxification and metabolism of xenobiotics and drugs (Mcquarters et al., 2014). The increase in cytochrome P450 transcript level suggested a positive response to 2.5 μL/L BT. Overall, ABCG2 and cytochrome P450 were vital for the survival of *B. cinerea* under BT stress. *Bcbrn1* (1,3,8-naphthalenetriol reductase), which is associated with melanin biosynthesis (Schumacher, 2016), was also increased throughout the experiment, suggesting possible melanization of the mycelia, and the current study has proven this hypothesis. In summary, BT activated the drug efflux, detoxification and secondary metabolite biosynthesis pathways of *B. cinerea*.

Phosphatidylserine decarboxylase (PSD) is responsible for the synthesis of phosphatidylethanolamine (PtdEtn) by decarboxylation of phosphatidylserine (PtdSer). Phosphatidylserine decarboxylase plays a central role in the phospholipid metabolism of prokaryotes and eukaryotes, and its catalytic product, PtdEtn, is a crucial phospholipid in mitochondria (Voelker, 1997) and plays an important role in full mitochondrial respiratory function (Birner et al., 2001; Gohil et al., 2005). Especially in the absence of fermentable carbon sources (e.g., glucose), the PtdEtn produced by PSD becomes essential (Birner et al., 2001). Thus, the increase in PSD transcription in the current study may suggest that the baseline glucose content of *B. cinerea* is not enough to support a defense against BT. Similarly, one gene (5,427,613) encoding a sugar transporter showed upregulation at all the exposure time points, suggesting a requirement for sugars as an energy source for *B. cinerea*. Overall, these results indicated that a larger energy supply was needed for *B. cinerea* in response to BT.

### Inhibition of Pectin Degradation Suggesting Low Utilization of Carbohydrate Sources in *B. cinerea*

The current study indicated that BT mainly affected the metabolism of *B. cinerea*, especially carbohydrate metabolism. “Pentose and glucuronate interconversions” and “starch and sucrose metabolism” were potential targeted pathways in *B. cinerea* in response to BT, suggesting that carbohydrate metabolism was required. The most significant differences were the inhibition of pectin degradation and activation of starch decomposition. In the pectin degradation process, four pectinases, including two PMEs (*Bcpme1* and *Bcpme2*) and two polygalacturonases (*Bcpgx1* and *Bcpg1*), which are components of cell-wall-degrading enzymes and important virulence factors (Valette-Collet et al., 2003; Manfredini et al., 2006), were significantly downregulated in *B. cinerea*. It is known that pectin demethylation by PMEs is helpful for depolymerization by endopolygalacturonases (endoPGs) (Lionetti et al., 2007); thus, it is coincidental that the expression of *Bcpme1*, *Bcpme2*, and *Bcpgx1* showed the same trend. The inhibition of pectin degradation suggested that BT had a major effect on the utilization of carbohydrate sources in *B. cinerea*.

### Activation of Five Signaling Pathways Regulating Glucose and Lipid Homeostasis in *B. odoriphaga* at 6 h

AMPK is a metabolic sensor and is usually activated under conditions of reduced intracellular ATP production (low energy) (Mihaylova and Shaw, 2011). It has been reported that mitochondrial inhibitors (e.g., biguanides and resveratrol) and starvation could increase AMP and ADP levels, thereby activating the AMPK signaling pathway (Mihaylova and Shaw, 2011; Garcia and Shaw, 2017). The AMPK signaling pathway plays an important role in increasing glucose uptake and fatty acid oxidation (Garcia and Shaw, 2017) and regulating gene transcription (Mihaylova and Shaw, 2011). It is possible that starvation caused by apastia (Zhao et al., 2016a) activated the AMPK signaling pathway, which indicated a lower energy condition and metabolic dysfunction.

The insulin signaling pathway was the second most enriched signaling pathway affected by BT; insulin is a vital metabolic hormone regulating carbohydrate and lipid metabolism in organisms. The majority of DEGs in this pathway were fatty acid synthases (FASs), indicating a dysregulation of fatty acid biosynthesis and metabolism. Other genes, such as insulin receptor (INSR), insulin receptor substrate 1 (IRS1), 3-phosphoinositide dependent protein kinase-1 (PDPK1), RAC serine/threonine-protein kinase (AKT) and PEPCK, were upregulated to regulate glucose homeostasis. Insulin signaling can influence feeding and locomotion behavior in insects such as *Drosophila* (Erion and Sehgal, 2013). Our previous study found that *B. odoriphaga* showed no ingestion or activity when treated with BT for 6 h (Zhao et al., 2016a). However, motivation of foraging is crucial for the survival of the insect, and insulin signaling could probably initiate starvation-induced food acquisition behavior (Zhao and Campos, 2012).

The adipocytokine signaling pathway includes the signaling cascades caused by adipocytokines (TNF-α, leptin and adiponectin), which are pivotal signaling molecules associated with insulin resistance (Borst, 2004; Zhao et al., 2013). In our study, we identified 17 upregulated genes (out of 20 total in this pathway), including IRS1 and tyrosine-protein phosphatase non-receptor type 11 (SHP2), suggesting increased glucose uptake and decreased insulin resistance (Rung et al., 2009; Ogunyemi et al., 2013). Similarly, in the KEGG enrichment analysis, insulin resistance was significantly changed. Upregulation of PEPCK could promote glucose production, and upregulation of INSR and IRS could stimulate glucose uptake. The GO enrichment analysis also showed that carbohydrate transport, such as glucose transport, was significantly activated when *B. odoriphaga* was exposed to BT for 6 h. In summary, BT improved glucose uptake and transport in *B. odoriphaga*, consistent with a previous study of BT derivatives (Pasternak et al., 2014).

FoxO transcription factors are important in regulating responses to many stimuli, such as insect diapause, stress resistance, nutritional adversity and energy homeostasis (Kramer et al., 2003; Cyclase et al., 2009; Poelchau et al., 2011). The activation of the FoxO signaling pathway suggested a positive response by *B. odoriphaga* involving the regulation of physiological processes. The fifth most changed pathway affected by BT was the PPAR signaling pathway; PPAR acts as a fat sensor to regulate the transcription of fat-metabolizing enzymes. Notably, the AMPK signaling pathway can influence the other four signaling pathways, and FoxO and PPAR can be regulated by the insulin, AMPK and adipocytokine pathways (Suzuki et al., 2007). Overall, these five signaling pathways interact with each other and mainly regulate glucose and lipid metabolism. The activation of these signaling pathways suggested that the energy and nutrient levels in *B. odoriphaga* fluctuated severely under BT treatment. Previous biochemical results have shown that BT decreases the levels of three nutrient types, proteins, glucose and lipids, in *B. odoriphaga* (Zhao et al., 2016b). Meanwhile, the Venn diagram shown in Supplementary Figure S3 suggested an important role of PEPCK in the signal transduction of *B. odoriphaga* in response to BT.

### Digestive System Alteration and Melanogenesis in *B. odoriphaga* at 24 h

Due to self-protection, *B. odoriphaga* refused to consume rhizomes exposed to the noxious and odorous compound BT. The above and previous results proved that low energy and glucose levels were present in *B. odoriphaga* after BT treatment for 6 h (Zhao et al., 2016b). In a state of starvation, insects adapt through risky behaviors, including searching for and consuming food under unfavorable conditions. We found that *B. odoriphaga* began to ingest the rhizomes at 24 h, thereby inducing alterations in the digestive system, including the “protein digestion and absorption,” “pancreatic secretion” and “salivary secretion” pathways. The associated digestive enzymes, especially trypsin, were greatly affected by BT, possibly leading to nutrient uptake impairment. As proteins are difficult to absorb through the lining of the small intestine, tryptic digestion is an essential component of protein absorption. Therefore, we can conclude that BT functions as a trypsin disrupter, interfering with protein absorption in *B. odoriphaga*.

In the glutathione metabolism pathway, we also found inhibition of 8 APN genes. In the small intestine, peptides from the hydrolysis of pancreatic and gastric proteases are finally digested with APN; therefore, the inhibition of APN again proved that BT affected protein absorption in *B. odoriphaga*. In addition, it has been proven that APN is a receptor for the *Bacillus thuringiensis* Cry1Ac toxin in *Spodoptera litura* and *Helicoverpa armigera* (Rajagopal et al., 2002; Sivakumar et al., 2007). Because BT is one of the secondary metabolites of *B. subtilis* (Liu et al., 2009a), we speculate that APN may be a potential target of BT. However, in the current study, the insect *B. odoriphaga* was treated with fumigation and not by feeding. Two reasons may explain these results: little BT residue was deposited on the rhizomes that were ingested by *B. odoriphaga*; there were still several upstream genes that regulated APN. Therefore, further studies are needed to verify this hypothesis, and we will investigate further in the future.

Furthermore, inhibition of tyrosine metabolism and melanogenesis were detected in our study, suggesting an inhibition of melanization in *B. odoriphaga*. Downregulation of 7 tyrosinase (also known as phenoloxidase, a rate-limiting enzyme for melanin biosynthesis) genes was responsible for the inhibition of the production of melanins, such as eumelanin. Moreover, tyrosinase is important for both developmental and defensive functions in insects, including melanization, sclerotization, wound healing, and parasite encapsulation; thus, tyrosinase inhibitors are being developed as alternative insecticides for controlling insect pests (Kim and Uyama, 2005). Given the vital role of tyrosinase in the immune defense mechanism of insects (Barnes and Siva-Jothy, 2000; Lee et al., 2008), its inhibition may cause decreased immunocompetence. Whether BT functions as a tyrosinase inhibitor still requires investigation.

### Similarities and Differences Between the Transcriptomic Profiles of the Fungus *B. cinerea* and the Insect Pest *B. odoriphaga*

The “pentose and glucuronate interconversions” pathway was altered in both the fungus *B. cinerea* and the insect *B. odoriphaga*, showing that carbohydrate metabolism was connected with the mode of action of BT in both *B. cinerea* and *B. odoriphaga*. However, the genes involved were different in the two species. In the insect *B. odoriphaga*, the UDP-glucuronosyltransferase (UGT; EC 2.4.1.17) and aldehyde reductase (AR; EC 1.1.1.21) genes accounted for a large percent of the DEGs participating in this pathway. UGTs are a group of phase II detoxification enzymes, and ARs are important in the accessory pathway of glucose metabolism, the polyol pathway. In contrast, in the fungus *B. cinerea*, the expression of pectin degradation enzyme genes such as *Bcpme1* and *Bcpgx1* was obviously decreased. Possible reasons for this difference are that the dose of the treatment was different (EC_50_ for *B. cinerea*; LC_30_ for *B. odoriphaga*), and the defense mechanisms of different species in response to the same exogenous compound are not necessarily the same. In summary, the most obvious commonality was that BT caused significant alterations in nutrient metabolism in both fungi and insects, and the “pentose and glucuronate interconversions” pathway was changed in both species.

One difference was that BT may act as a melanogenesis disrupter for the insect but as an activator for the fungus. In fungi, the increased melanin biosynthesis may have been based on compensation for other normal growth processes, such as carbohydrate metabolism. This may reflect the fact that an organism exposed to an exogenous compound shifts the balance among its potential physiological processes when energy conflict arises, supporting one process at the expense of another. However, melanogenesis is an immune defense mechanism in insects, and its decrease suggests an unfavorable growth condition in insects, which negatively affects the subsequent developmental process. Overall, when encountering adversity, the principles of resource allocation differ in different species.

## Conclusion

The transcriptome profiles of the fungus *B. cinerea* and the insect pest *B. odoriphaga*, in response to BT, were compared in the current study. The evidence suggested that BT affected nutrient metabolism, especially carbohydrate transport, in both species, and the “pentose and glucuronate interconversions” pathway was altered in both species. The insect showed a more complicated response, with several signaling pathways and the digestive system functioning together to regulate glucose and lipid homeostasis. PEPCK was the core gene in the signal transduction of *B. odoriphaga* in response to BT. In addition, we identified several potential insecticidal target genes, such as trypsin, APN, and tyrosinase. Furthermore, BT may act as a melanogenesis disrupter for the insect but as an activator for the fungus. Exploring the specific mechanisms of action of BT is a challenging and complicated process. More studies are needed to evaluate whether a common target exists in different species.

## Data Availability Statement

The raw Illumina sequencing reads have been deposited in the NCBI SRA database with the accession numbers SRP174462 for *Botrytis cinerea* and SRP174932 for *Bradysia odoriphaga*.

## Author Contributions

KC, YZ, and FL conceived and designed the study. YZ, JD, and LH carried out the experiments and analyzed the data. KC, BL, and WM assisted with the bioinformatic analysis. KC and FL wrote and revised the manuscript.

## Conflict of Interest

The authors declare that the research was conducted in the absence of any commercial or financial relationships that could be construed as a potential conflict of interest.

## Footnotes

1 https://www.ncbi.nlm.nih.gov/Structure/cdd/wrpsb.cgi

## Supplementary Material

FIGURE S1 Venn diagram of functional unigene annotations of the *Bradysia odoriphaga* transcriptome based on five major public databases (NR, InterPro, Swiss-Prot, COG, and KEGG).

FIGURE S2 Distribution of the annotated species by BLAST analysis of *Bradysia odoriphaga* sequences against the Nr database.

FIGURE S3 Venn diagram of the DEGs in five signaling pathways at 6 h in *Bradysia odoriphaga*, highlighting core signaling genes.

TABLE S1 Primers used in the current study for RT-qPCR.

TABLE S2 Summary of RNA-seq reads mapped to the *Botrytis cinerea* B05.10 genome.

TABLE S3 Differentially expressed genes identified in *Botrytis cinerea* exposed to benzothiazole for 12, 24 and 48 h.

TABLE S4 The 28 core genes shared by the 12, 24 and 48 h comparison groups (Figure 3B) identified in the *Botrytis cinerea* transcriptome.

TABLE S5 Detailed information for the overlapping DEGs involved in the two pathways shared by the 24 and 48 h groups (Figure 5B) in the *Botrytis cinerea* transcriptome.

TABLE S6 Summary statistics of the clean reads and assembled unigenes of the *Bradysia odoriphaga* transcriptome.

TABLE S7 Annotations of unigenes from seven public databases.

TABLE S8 Differentially expressed genes identified in *Bradysia odoriphaga* exposed to benzothiazole for 6 and 24 h.

TABLE S9 Detailed information for the KEGG enrichment analysis of the DEGs in the *Bradysia odoriphaga* transcriptome.

## Abbreviations

ABC: ATP-binding cassette
AKT: RAC serine/threonine-protein kinase
AMPK: AMP-activated protein kinase
APN: aminopeptidase N
AR: aldehyde reductase
Bcbrn1: 1,3,8-naphthalenetriol reductase
BP: biological process
BT: benzothiazole
CC: cellular component
COEs: carboxylesterases
COG: Clusters of Orthologous Groups of proteins
CON: control
DEGs: differentially expressed genes
DHN: dihydroxynaphthalene
endoPGs: endopolygalacturonases
FASs: fatty acid synthases
FoxO: forkhead box class O
FPKM: fragments per kilobase of transcript per million mapped reads
GO: Gene Ontology
GSTs: glutathione-S transferases
INSR: insulin receptor
IRS1: insulin receptor substrate 1
KEGG: Kyoto Encyclopedia of Genes and Genomes
MF: molecular function
PEPCK: phosphoenolpyruvate carboxykinase
NGS: next-generation sequencing
Nr: NCBI non-redundant protein sequences
Nt: NCBI non-redundant nucleotide sequences
OAADPr: 2′-O-acetyl-ADP-ribose
P450s: cytochrome P450s
PDA: Potato dextrose agar
PPAR: peroxisome proliferator-activated receptor
PSD: phosphatidylserine decarboxylase
PtdEtn: phosphatidylethanolamine
PtdSer: phosphatidylserine
PDPK1: 3-phosphoinositide dependent protein kinase-1
RPS15: ribosomal protein S15
RT-qPCR: reverse transcription quantitative PCR
SHP2: tyrosine-protein phosphatase non-receptor type 11
SRA: Sequence Read Archive
Sir2: silent information regulator 2
TEM: transmission electron microscopy
UGT: UDP-glucuronosyltransferase.
