# Supplementary figures and images for: Comparison of Transcriptome Profiles of the Fungus Botrytis cinerea and Insect Pest Bradysia odoriphaga in Response to Benzothiazole
Source: Front Microbiol. 2020 Jun 12;11:1043. doi: 10.3389/fmicb.2020.01043 (PMC7325989; doi:10.3389/fmicb.2020.01043)

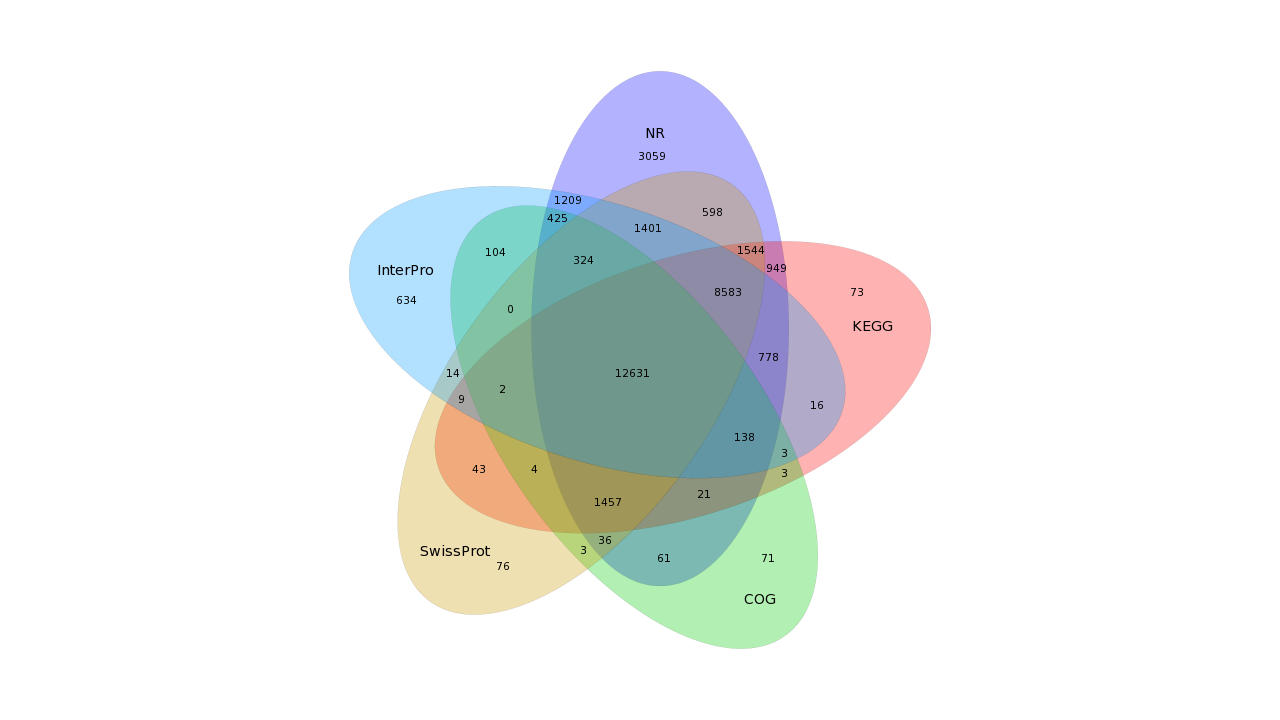

Supplement: FIGURE S1 — Venn diagram of functional unigene annotations of the Bradysia odoriphaga transcriptome based on five major public databases (NR, InterPro, Swiss-Prot, COG, and KEGG). [file Image_1.TIF]

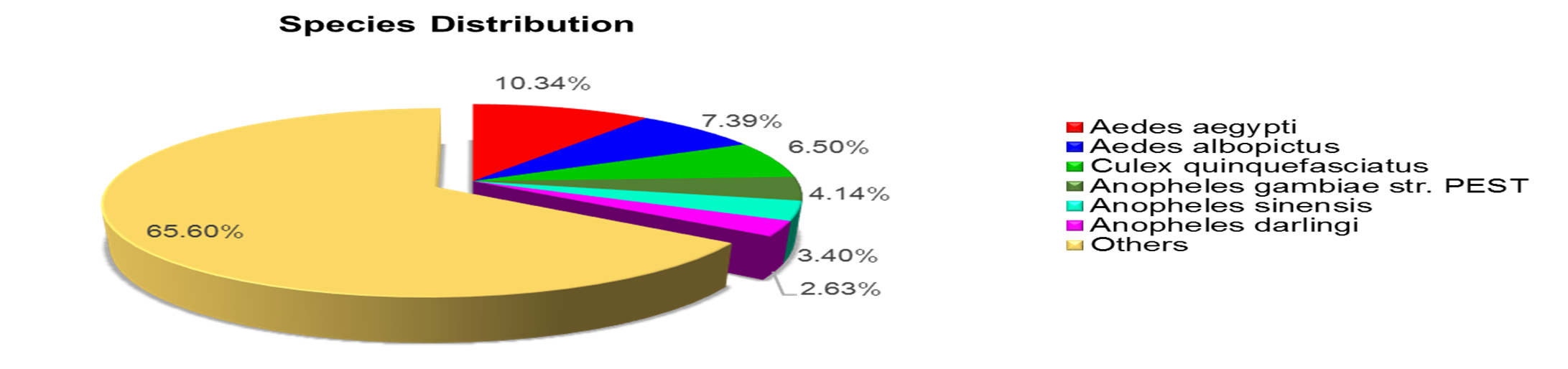

Supplement: FIGURE S2 — Distribution of the annotated species by BLAST analysis of Bradysia odoriphaga sequences against the Nr database. [file Image_2.TIF]

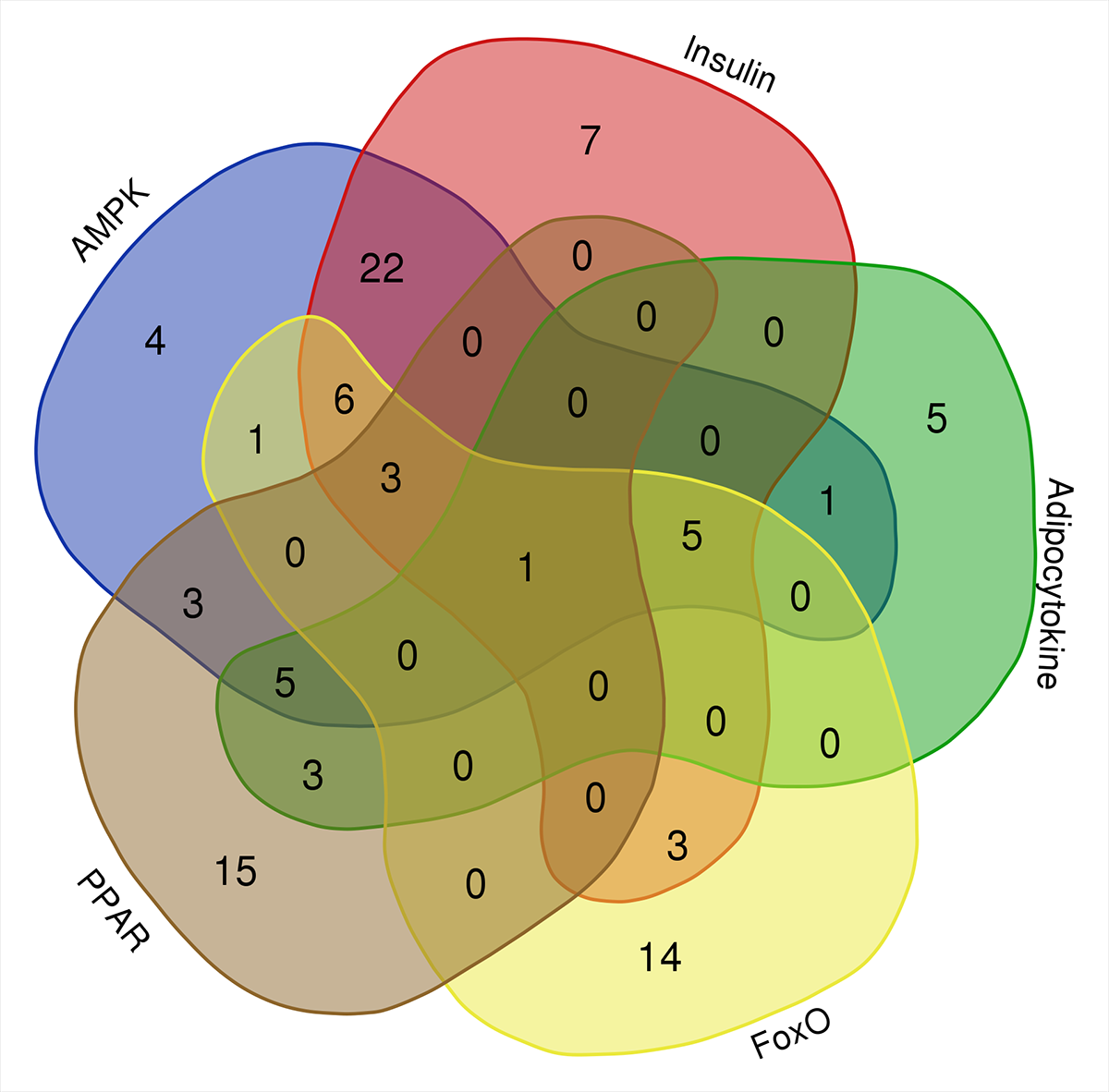

Supplement: FIGURE S3 — Venn diagram of the DEGs in five signaling pathways at 6 h in Bradysia odoriphaga, highlighting core signaling genes. [file Image_3.TIF]
